# Transgenic Mice Overexpressing Human Alpha-1 Antitrypsin Exhibit Low Blood Pressure and Altered Epithelial Transport Mechanisms in the Inactive and Active Cycles

**DOI:** 10.3389/fphys.2021.710313

**Published:** 2021-09-22

**Authors:** Lauren P. Liu, Mohammed F. Gholam, Ahmed Samir Elshikha, Tamim Kawakibi, Nasseem Elmoujahid, Hassan H. Moussa, Sihong Song, Abdel A. Alli

**Affiliations:** ^1^Department of Physiology and Functional Genomics, University of Florida College of Medicine, Gainesville, FL, United States; ^2^Department of Pathology, Immunology and Laboratory Medicine, University of Florida College of Medicine, Gainesville, FL, United States; ^3^Department of Pharmaceutics, University of Florida College of Medicine, Gainesville, FL, United States; ^4^Division of Nephrology, Hypertension, and Renal Transplantation, Department of Medicine, University of Florida College of Medicine, Gainesville, FL, United States

**Keywords:** hAAT, blood pressure, electrolytes, kidney, epithelial Na^+^ channel

## Abstract

Human alpha-1 antitrypsin (hAAT) is a versatile protease inhibitor, but little is known about its targets in the aldosterone-sensitive distal nephron and its role in electrolyte balance and blood pressure control. We analyzed urinary electrolytes, osmolality, and blood pressure from hAAT transgenic (hAAT-Tg) mice and C57B/6 wild-type control mice maintained on either a normal salt or high salt diet. Urinary sodium, potassium, and chloride concentrations as well as urinary osmolality were lower in hAAT-Tg mice maintained on a high salt diet during both the active and inactive cycles. hAAT-Tg mice showed a lower systolic blood pressure compared to C57B6 mice when maintained on a normal salt diet but this was not observed when they were maintained on a high salt diet. Cathepsin B protein activity was less in hAAT-Tg mice compared to wild-type controls. Protein expression of the alpha subunit of the sodium epithelial channel (ENaC) alpha was also reduced in the hAAT-Tg mice. Natriuretic peptide receptor C (NPRC) protein expression in membrane fractions of the kidney cortex was reduced while circulating levels of atrial natriuretic peptide (ANP) were greater in hAAT-Tg mice compared to wild-type controls. This study characterizes the electrolyte and blood pressure phenotype of hAAT-Tg mice during the inactive and active cycles and investigates the mechanism by which ENaC activation is inhibited in part by a mechanism involving decreased cathepsin B activity and increased ANP levels in the systemic circulation.

## Introduction

Alpha-1-antitrypsin (AAT) is a serine protease inhibitor (SERPIN), which is mainly produced in the liver and secreted in to the circulation (Kohnlein and Welte, 2008). The well-known function of AAT is to inhibit neutrophil elastase (NE). Deficiency of AAT leads to higher activity of NE and chronic obstructive pulmonary disease (COPD) in humans. Increasing evidence show that AAT is also a multifunctional protein with anti-inflammatory and immunoregulatory properties in addition to its inhibitory function of proteinases (Bai et al., 2021). It has been shown that human AAT (hAAT) has therapeutic potential in mouse models of human diseases including type 1 diabetes (Song et al., 2004; Lu et al., 2006; Ma et al., 2010), arthritis (Grimstein et al., 2010, 2011), lupus (Elshikha et al., 2016, 2018), and osteoporosis (Akbar et al., 2016). It has also been shown that AAT has anti-aging effect (Yuan et al., 2018). These results suggest that AAT may have more unknown functions.

Scharpe et al. (1976) was the first group to report AAT as an endogenous inhibitor of renin. Renin is the rate limiting step in the Renin-Angiotensin-Aldosterone system (RAAS). The aldosterone-sensitive nephron includes the sodium chloride co-transporter (NCC) and the epithelial sodium channel (ENaC). Both NCC and ENaC contribute to total body sodium balance and blood pressure control (Verouti et al., 2015). The latter is positively regulated by a myriad of proteases including furin (Hughey et al., 2004), channel-activating protease 2 (CAP2) (Garcia-Caballero et al., 2008), kallikrein (Patel et al., 2012), and the cysteine protease cathepsin B (Alli et al., 2012b) and cathepsin S (Haerteis et al., 2012).

In addition to the RAAS, the natriuretic peptide system also contributes to sodium and blood pressure regulation. Early studies suggested atrial natriuretic peptide (ANP) is a potent inhibitor of renin release (Nishikimi et al., 2006) and aldosterone secretion (Ganguly, 1992). However, the molecular mechanisms and mediators that are involved are still being investigated. Natriuretic peptides serve many physiological functions including promoting vasodilation, natriuresis, and diuresis, and reducing blood volume and pressure (Nishikimi et al., 2006). Natriuretic peptides inhibit ENaC activity in the aldosterone sensitive distal nephron (Guo et al., 2013). The binding of ANP and B-type natriuretic peptide (BNP) to the guanyl cyclase coupled cell surface receptors GCA and GCB (NPRA and NPRB) result in the generation of the second messenger cGMP (Koller and Goeddel, 1992). The natriuretic peptide clearance receptor NPRC regulates the availability of natriuretic peptides by internalization and degradation of the bound ligands (Anand-Srivastava et al., 1996). NPRC is also coupled to the activation of phospholipase C (Anand-Srivastava, 1997), inhibition of adenylyl cyclase (Mouawad et al., 2004), and calcium mobilization (Alli and Gower, 2009). Posttranslational modifications including phosphorylation are believed to regulate the physiological actions of NPRC (Alli and Gower, 2010). A previous study showed NPRA is mainly involved in the natriuretic peptide dependent regulation of renal ENaC as cGMP effectively inhibits renal ENaC activity (Guo et al., 2013). But it is likely that other signaling pathways involving cGMP and nitric oxide (NO) contribute to this mechanism since soluble guanylyl cyclase (sGC) inhibitors reduce the effect of ANP on ENaC activity (Guo et al., 2013). The activation sGC occurs through the binding of nitric oxide (NO) (Krishnan et al., 2018).

A number of epithelial transport mechanisms in the kidney that contribute to electrolyte and blood pressure regulation have been shown to be governed by circadian rhythms. The circadian clock consists of the CLOCK, Cryptochrome (Cry1 and −2), Period (Per1-3), and Bmal1 (Solocinski and Gumz, 2015). SiRNA mediated knockdown of the circadian clock protein Per1 has been shown to result in the decrease in renal NCC (Richards et al., 2014) and ENaC activity (Alli et al., 2019). The regulation of NCC and ENaC has been shown to occur at the transcriptional and protein levels (Richards et al., 2014; Alli et al., 2019). Although both renal NCC and ENaC have been previously shown to be regulated in a circadian dependent manner, the circadian regulation of proteases that activate ENaC has not been studied.

At least two isoforms in the cathepsin family of cysteine proteases have been shown to cleave and activate ENaC. Abnormally high ENaC activity leads to sodium retention and hypertension. Here we tested the hypothesis that overexpression of AAT lowers cathepsin B activity in the kidney leading to less proteolysis of ENaC and lower blood pressure. Cathepsins are known to cleave a myriad of proteins including actin cytoskeleton proteins that regulate membrane expression of transporters such as NCC and receptors such as NPRC in the kidney that contribute to electrolyte balance and blood pressure regulation (Keisuke et al., 2020). Thus, we also investigated whether AAT overexpression could alter renal NCC and NPRC protein expression. To our knowledge, this is the first study investigating the regulation of electrolytes and blood pressure as well as epithelial transport mechanisms in hAAT-Tg mice compared to wild-type control mice. Another goal of this study was to investigate changes in electrolytes and blood pressure in the active and inactive cycles of transgenic hAAT overexpressing mice. The inactive cycle represents the first 12-h part of the day (the AM) whereas the active cycle represents the second 12-h part of the day (the PM). Accumulating studies have shown that a non-dipping blood pressure phenotype during the inactive cycle leads to an increased risk of cardiovascular disease, stroke, and high mortality (Solocinski et al., 2017), therefore we investigated whether epithelial transport methods in the aldosterone-sensitive distal nephron can contribute to changes in electrolytes and blood pressure in mice overexpressing hAAT.

## Materials and Methods

### Animals

All animal studies were performed under a protocol approved by the University of Florida’s Institutional Animal Care and Use Committee and the studies were in compliance with the National Institutes of Health *Guide for the Care and Use of Laboratory Animals*. hAAT-Tg mice were developed and maintained at the University of Florida as described by Elshikha et al. (2019). The C57B/6 mice and the hAAT-Tg mice were age matched at 3–5 months at the start of the study.

### Electrolyte and Osmolality Measurements

0.1 ml of urine collected from mice metabolic cage studies were centrifuged at 13,000°×°g for 6 min. The samples were made into a solution containing 1-part urine and 3-parts ultrapure H_2_O then into another solution containing the 1-part urine mix and 2-parts urine diluent (Diamond Diagnostics, Holliston, MA) before being thoroughly mixed. The second solution, 1-part urine mix and 2-parts urine diluent, was aspirated by the SmartLyte machine (SmartLyte, Diamond Diagnostics). Before being aspirated, the SmartLyte analyzer was calibrated for measurement quality assurance. Then, the concentrations of the urinary electrolyte sodium, potassium, and chloride were measured. Urine osmolality was measured through an auto-sampling turntable model 2430 osmometer (Precisions Systems Inc.) where 30 μl of each sample was analyzed per minute. The osmometer utilizes the freezing point depression method to accurately indicate the total concentration of solutions.

### Blood Pressure Measurements

The mice were subjected to a 3-day acclimation period and then maintained on a normal salt diet (0.40% NaCl) (Teklad, Envigo) for 3 days. Baseline blood pressure measurements were taken while the mice were on a normal salt diet. The mice were then switched to a high salt diet (4.0% NaCl) (Teklad, Envigo), where their blood pressure measurements were taken again. Blood pressure was measured in the AM (8 am–10 am) and in the PM (8 pm–10 pm) by the tail cuff method (IITC MRBP System from Life Science Inc.). Data was analyzed using the MRBP Software. Systolic blood pressure reference was defined according to the AHA.

### Blood Sample Collection

Blood was drawn from the mice during both the normal salt and high salt phases, once during the active cycle and once during the inactive cycle. The mice were put into restrainers and their tail veins were punctured using 18-gauge sterile hypodermic needles. The blood was collected into a capillary tube where it was then transferred to a microcentrifuge tube for storage.

### Human AAT Specific ELISA

Detection of hAAT in hAAT-Tg mice compared to C57B/6 wild-type mice was performed as previously described by Elshikha et al. (2019). Briefly, a microtiter plate was coated with goat anti-hAAT (Bethyl, Montgomery, TX, United States) in Voller’s buffer overnight at 4°C and was blocked with 3% BSA for 1 h at 37°C. Duplicate standard curves and samples were incubated at 37°C for 1 h. Rabbit anti-hAAT (Sigma-Aldrich, St. Louis, MO, United States) was reacted with the captured antigen at 37°C for 1 h A third antibody, goat anti-rabbit IgG conjugated with peroxidase (Sigma-Aldrich, St. Louis, MO, United States) was incubated at 37°C for 1 h.

### Tissue Preparation

Twenty-five milligram sections of the renal cortex or medulla were homogenized using an Omni TH homogenizer (Warrenton, VA) in 500 μl tissue protein extraction reagent (TPER) (Thermo Fisher Scientific; Waltham, MA). The tissue lysate was incubated on ice for 30 min while mixing every 10 min and then centrifuged for 10 min at 13,000 rpm at 4°C in a benchtop centrifuge (Thermo IEC). The supernatant was subject to ultracentrifugation for 30 min at 34,000 rpm using a SW55.1 rotor (Beckman). The supernatant was labeled “soluble fraction” and stored at −80°C. The pellet was reconstituted in 250 μl TPER before being sonicated for two 5 second intervals and then labeled as “membrane fraction” and stored at −80°C. Protein concentrations of the soluble and membrane fractions for the renal cortex and medulla lysates were determined by a BCA protein assay (Thermo Fisher Scientific).

### Western Blotting

Kidney tissue samples were homogenized and then the total protein concentrations of these samples were determined through BCA (bicinchoninic acid) protein assays (Thermo Fisher Scientific). After total protein concentrations were determined, the samples were loaded into 20-well, 4–20% Tris–HCl polyacrylamide gels. The gels were inserted into a Criterion electrophoresis system (Bio-Rad) that resolved the proteins. These resolved proteins were then transferred onto a nitrocellulose membrane (GE Healthcare, Piscataway, NJ) using a Criterion transfer system (Bio-Rad). When the transfer completed, the membranes were blocked in a 5% non-fat milk 1°×°TBS (Bio-Rad; 1706435) solution at room temperature on an automated rocker for an hour. After blocking, the nitrocellulose membranes were washed twice with 1°×°TBS and incubated [NCC (Thr53) Antibody (1:1,000) (PhosphoSolutions; p1311-53), beta Actin Monoclonal HPR Antibody (1:10,000) (Thermo Fisher Scientific; MA5-15739-HRP), ENaC Alpha Antibody (1:1,000) (Alli et al., 2012a), Cathepsin B Antibody (1:1,000) Cell Signaling; 31718), Anti-Kallikrein 1/KLK1 Antibody (PA1709) (1:1,000) (Boster Bio, Pleasanton, CA; PA1709), NPRC Antibody (1:1,000) (Alli and Gower, 2009, 2010), ENaC Beta (1:1000) (Alli et al., 2012a), and ENaC Gamma (1:1,000) (Malik et al., 2001)] on an automated rocker for a minimum of 24 h (maximum of 48 h) at 4°C. The next day, the membranes were washed a total of three times with 1°×°TBS before being incubated for an hour with horseradish peroxidase-conjugated goat anti-rabbit secondary antibody at a 1:3,000 dilution prepared in blocking solution. After the completion of the incubation, the nitrocellulose membranes were washed with 1°×°TBS four times and then incubated with ECL Select Western Blotting Detection Reagent (GE Healthcare) for 6 min. The nitrocellulose membranes were then imaged on a Bio-Rad imager.

### Cathepsin-B Activity Assay

Cathepsin B activity from kidney cortex or medulla lysates was measured using a cathepsin B fluorometric activity assay (Abcam; Cambridge, MA) according to the manufacturer instructions but with the following modifications: twenty-five micrograms of protein was prepared to a final volume of 50 μl using lysis buffer and then added to separate wells of a 96 well plate. Next, 50 μl of cathepsin-B (CB) reaction buffer was added followed by 2 μl of 10 mM CB substrate (Ac-RR-AFC) (200 μM final concentration). As a negative control, 2 μl of CB inhibitor was added to a pooled sample for each group. The plate was incubated at 37°C for 90 min while being protected from light. The samples were then measured on a fluorescent microplate reader at Ex/Em = 400/505 nm. The background signal (buffer only) was subtracted from the sample readings before fold-increase or fold-decrease in cathepsin-B activity was determined by comparing the relative fluorescence units from each group.

### ANP ELISA

ANP concentration in blood samples collected from each mouse during both the normal salt and high salt phases, once during their inactive cycle and once during their active cycle, was measured using the ANP ELISA Kit (NPPA) (Abcam).

### Nitric Oxide Measurements

A nitric oxide assay (Thermo Fisher Scientific) was used to quantitatively measure nitrate and nitrite concentrations in mouse blood samples. For the nitrate assay, a 1:10 dilution of each blood sample was prepared to a final volume of 50 μl in reagent diluent. Next, 25 μl of diluted NADH and then the same amount of nitrate reductase was added to each 50 μl sample in a 96 well plate. The assay was then incubated at 37°C for 30 min before Greiss reagent I and Greiss reagent II were added to the assay. The plate was mixed by gentle shaking and then incubated for 10 min at room temperature before the optical density (OD) was read at 540 nm. For the nitrate assay, a 1:10 dilution of each blood sample was prepared to a final volume of 50 μl in reagent diluent. Next 50 μl of Griess reagent I and then the same amount of Griess reagent II were added to each 50 μl sample in a 96 well plate. The plate was mixed by gentle shaking and then incubated at room temperature for 10 min before the optical density was read at 540 nm.

### Statistical Analysis

To analyze the statistical significance of the data collected, we performed a One-Way ANOVA followed by a Holm-Sidak comparison if multiple groups were being compared. If only two groups were being compared, we performed a Student’s *t*-test. If there was no difference between the males and females when compared, we pooled the data for both sexes to only compare the differences between the strains of mice during the different activity cycles. We used a significance level of *P* < 0.05.

## Results

### hAAT-Tg Mice Have Lower Urinary Electrolyte Concentrations and Lower Urinary Osmolality Compared to Wild-Type C57B/6 Mice

A transgenic mouse line over expressing hAAT (hAAT-Tg) has been developed and back crossed with C57BL/6 mice. Since CMV enhancer and chicken-beta-actin promoter was used, these animals express hAAT in most of the organs including kidney (Elshikha et al., 2019). We have shown that hAAT-Tg mice are resistant to pristane-induced diffuse alveolar hemorrhage (DAH) (Elshikha et al., 2019). In this study, we employ hAAT-Tg mouse model and use C57BL/6 as a control to test the effect of hAAT on hypertension. First, we confirmed the level of hAAT expression in hAAT-Tg mice during the inactive (AM) and active cycles (PM) using a hAAT specific ELISA, which does not detect mouse AAT (Supplementary Figure 1).

**FIGURE 1:**
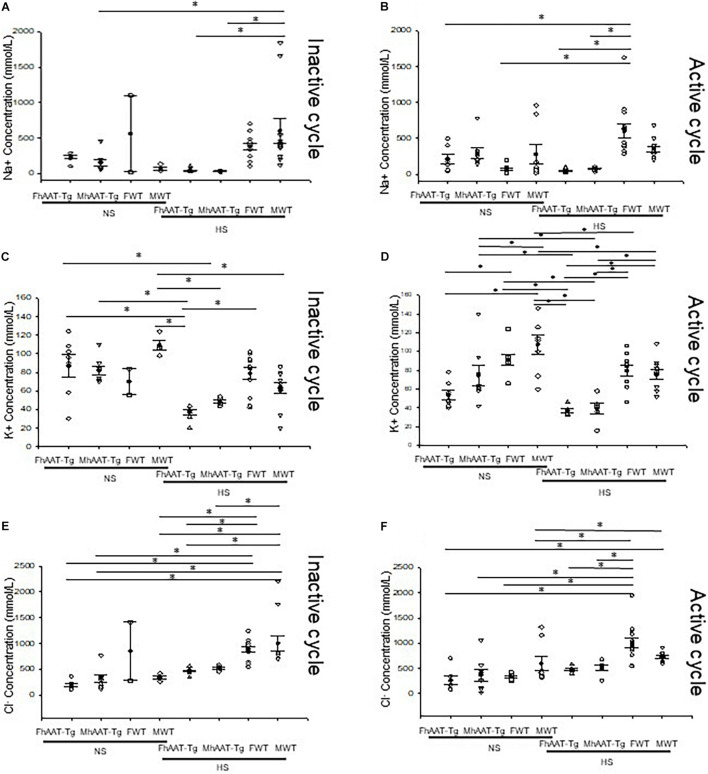
Urinary electrolytes corresponding to the inactive and active cycles of male and female hAAT-Tg and C57B/6 wild-type mice maintained on a normal or high salt diet. **(A)** Urinary sodium concentrations during the inactive cycle for female and male hAAT-Tg mice and C57B/6 wild-type mice maintained on a normal salt (NS) or high salt (HS) diet. **(B)** Urinary sodium concentrations during the active cycle for female and male hAAT-Tg mice and C57B/6 wild-type mice maintained on a normal salt (NS) or high salt (HS) diet. **(C)** Urinary potassium concentrations during the inactive cycle for female and male hAAT-Tg mice and C57B/6 wild-type mice maintained on a normal salt (NS) or high salt (HS) diet. **(D)** Urinary potassium concentrations during the active cycle for female and male hAAT-Tg mice and C57B/6 wild-type mice maintained on a normal salt (NS) or high salt (HS) diet. **(E)** Urinary chloride concentrations during the inactive cycle for female and male hAAT-Tg mice and C57B/6 wild-type mice maintained on a normal salt (NS) or high salt (HS) diet. **(F)** Urinary chloride concentrations during the active cycle for female and male hAAT-Tg mice and C57B/6 wild-type mice maintained on a normal salt (NS) or high salt (HS) diet. *N* = 7 hAAT-Tg (4 female and 3 male) and *N* = 8 C57B/6 (4 female and 4 male) mice. These results were analyzed using a One-Way ANOVA followed by a Holm-Sidak comparison. We used a significance level of *p < 0.05.

The kidneys play an important role in regulating total body electrolyte balance. If hAAT has an effect on kidney function, urinary electrolyte concentration will be affected. Therefore, we measured electrolytes in both the inactive and active cycles of female and male hAAT-Tg and C57B/6 wild-type mice while maintained on either a normal or high salt diet (Supplementary Figure 2). Urinary concentrations of sodium were significantly lower in male hAAT-Tg mice compared to male wild-type control mice maintained on a high salt diet for the inactive cycle while the urinary concentrations of sodium were significantly lower in female hAAT-Tg mice compared to female wild-type mice maintained on a high salt diet for the active cycle (Figures 1A,B). Urinary concentrations of potassium were significantly lower in female hAAT-Tg mice compared to female wild-type mice maintained on a high salt diet for the inactive cycle while the urinary concentrations of potassium were significantly lower for female and male hAAT-Tg mice compared to female and male wild-type mice maintained on a high salt diet for the active cycle (Figures 1C,D). Similarly, urinary concentrations of chloride were significantly lower in female and male hAAT-Tg mice compared to female and male wild-type mice maintained on a high salt diet for the inactive cycle while the urinary concentrations of chloride were significantly lower for female hAAT-Tg mice compared to female wild-type mice maintained on a high salt diet for the active cycle (Figures 1E,F). These results suggest that hAAT has an effect on urinary electrolyte concentrations and kidney functions.

**FIGURE 2:**
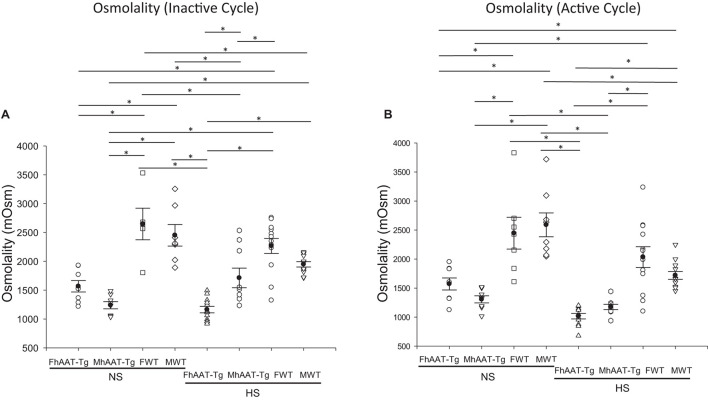
Urine osmolality for male and female hAAT-Tg and C57B/6 wild-type mice maintained on a normal or high salt diet during the inactive and active cycles. **(A)** Urinary osmolality during the inactive cycle for female and male hAAT-Tg mice and C57B/6 wild-type mice maintained on a normal salt (NS) or high salt (HS) diet. **(B)** Urinary osmolality during the active cycle for female and male hAAT-Tg mice and C57B/6 wild-type mice maintained on a normal salt (NS) or high salt (HS) diet. *N* = 7 hAAT-Tg (4 female and 3 male) and *N* = 8 C57B/6 (4 female and 4 male) mice. These results were analyzed using a One-Way ANOVA followed by a Holm-Sidak comparison. We used a significance level of *p < 0.05.

Since the kidneys also play an important role in regulating urine osmolality, we next, investigated urinary osmolality in both the inactive and active cycles of hAAT-Tg and C57B/6 wild-type mice (Supplementary Figure 3). For the inactive cycle, urine osmolality was significantly less in the male hAAT-Tg mice compared to male wild-type mice maintained on a normal salt diet while urine osmolality was significantly less in the female hAAT-Tg mice compared to female wild-type mice maintained on a high salt diet (Figure 2A). For the active cycle, female hAAT-Tg mice had a significantly lower urine osmolality compared to female wild-type mice maintained on a normal salt diet while female and male hAAT-Tg mice had significantly lower urine osmolality compared to female and male wild-type mice, respectively (Figure 2B). These results indicate that hAAT has an effect on urine osmolality consistent with its effect on urine electrolyte concentration.

### hAAT-Tg Mice Exhibit Lower Systolic Blood Pressure Compared to Wild-Type C57B/6 Mice in the Inactive and Active Cycles

**FIGURE 3:**
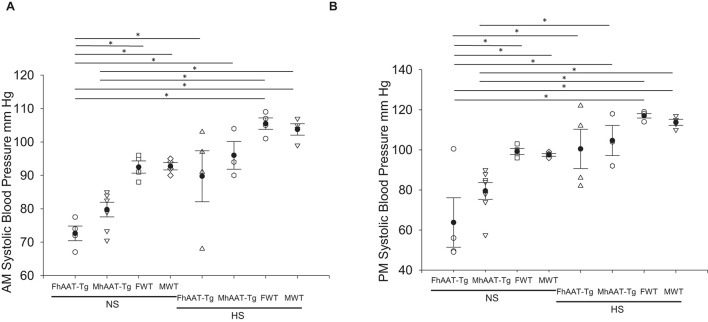
Systolic blood pressure of male and female hAAT-Tg and C57B/6 wild-type mice maintained on a normal or high salt diet during the inactive and active cycles. **(A)** Tail cuff systolic blood pressure measurements during the inactive (AM) cycle for female and male hAAT-Tg mice and C57B/6 wild-type mice maintained on a normal salt (NS) or high salt (HS) diet. **(B)** Tail cuff systolic blood pressure measurements during the active (PM) cycle for female and male hAAT-Tg mice and C57B/6 wild-type mice maintained on a normal salt (NS) or high salt (HS) diet. *N* = 10 hAAT-Tg (4 female and 6 male) and *N* = 8 C57B6 (4 female and 4 male) mice. These results were analyzed using a One-Way ANOVA followed by a Holm-Sidak comparison. We used a significance level of *p < 0.05.

In addition to maintaining electrolyte balance and regulating urine osmolality, the kidneys also contribute to the regulation of blood pressure. Therefore, we measured systolic blood pressure in the inactive and active cycles of hAAT-Tg and C57B/6 wild-type mice while maintained on either a normal or high salt diet (Supplementary Figure 4). Systolic blood pressure was significantly lower in female hAAT-Tg mice compared to female wild-type mice maintained on a normal salt diet for the inactive cycle and active cycle (Figures 3A,B). There may be a slight underestimation of the blood pressure measurements for both groups although the mice were trained to the machine.

### No Change in Cathepsin B Expression in hAAT-Tg Mice Compared to Wild-Type Control Mice

**FIGURE 4:**
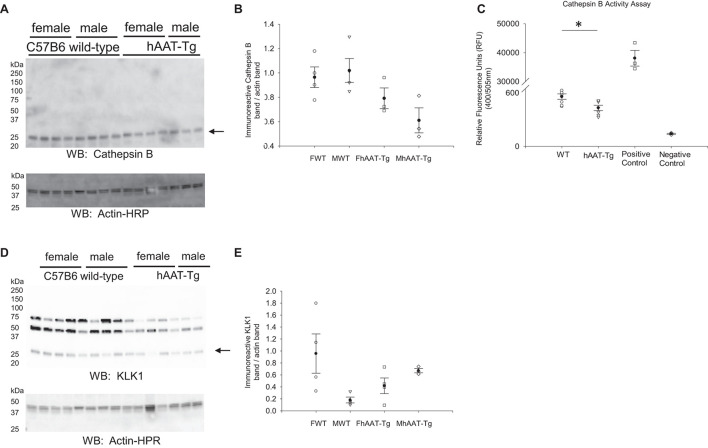
Western blot analysis of cathepsin B and kidney kallikrein protein expression from the soluble fraction of kidney cortex lysates. **(A)** Western blot analysis of the cathepsin B protein expression from the soluble fraction of kidney cortex lysates harvested from female and male hAAT-Tg mice and C57B/6 wild-type mice. **(B)** Densitometric analysis of the immunoreactive cathepsin B band in panel **(A)** normalized to actin. Lane 11 of the cathepsin B blot and the corresponding actin blot was omitted from the analysis. **(C)** Cathepsin B activity assay showing endogenous cathepsin B activity in kidney cortex lysates of wild-type and hAAT-Tg mice. **(D)** Western blot analysis of the kallikrein protein expression from the soluble fraction of kidney cortex lysates harvested from female and male hAAT-Tg mice and C57B/6 wild-type mice. **(E)** Densitometric analysis of the immunoreactive kallikrein band in panel **(D)** normalized to actin. *N* = 7 hAAT-Tg (4 female and 3 male) and *N* = 8 C57B/6 (4 female and 4 male) mice maintained initially on a normal salt diet and then a high salt diet and euthanized in the AM. These results were analyzed using a One-Way ANOVA followed by a Holm-Sidak comparison and a Student’s *t*-test. We used a significance level of *p < 0.05.

Cathepsin B is one of many proteases that is known to cleave and activate renal ENaC (Larionov et al., 2019). Since hAAT may inhibit cathepsin B activity, we investigated whether cathepsin B protein expression and activity are attenuated in hAAT overexpressing mice compared to wild-type control mice (Irving et al., 2002). Western blot and densitometric analysis showed no significant change in cathepsin B protein expression (Figures 4A,B). Cathepsin B activity however was significantly less in the hAAT-Tg mice compared to the wild-type control mice (Figure 4C and Supplementary Figure 5).

Kidney kallikrein is also known to contribute to the proteolytic activation of renal ENaC and this protease is also inhibited by hAAT (Scott et al., 1986). Therefore, we investigated differences in kallikrein expression between the two groups. As shown in Figures 4D,E, there were no significant differences between the groups.

### Increased Membrane Protein Expression of Renal pNCC in Female hAAT-Tg Mice Compared to Male hAAT-Tg and C57B/6 Wild-Type Mice

**FIGURE 5:**
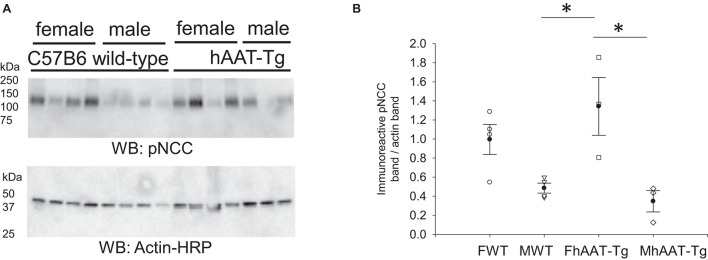
Western blot analysis of phospho-NCC protein expression from the membrane fraction of kidney cortex lysates. **(A)** Western blot analysis of the alpha subunit of the active membrane bound form of phosphorylated sodium chloride cotransporter (pNCC) protein expression from the membrane fraction of kidney cortex lysates harvested from female and male hAAT-Tg mice and C57B/6 wild-type mice after being maintained initially on a normal salt diet and then a high salt diet and euthanized in the AM. *N* = 7 hAAT-Tg (4 female and 3 male) and *N* = 8 C57B/6 (4 female and 4 male) mice. **(B)** Densitometric analysis of the immunoreactive pNCC bands in panel **(A)** normalized to actin. Lane 11 of the pNCC blot and the corresponding actin blot was omitted from the analysis. These results were analyzed using a One-Way ANOVA followed by a Holm-Sidak comparison. We used a significance level of *p < 0.05.

NCC is associated with the apical plasma membrane of the aldosterone-sensitive distal nephron (Moes et al., 2014). Decreased or increased activity of renal NCC is associated with hypotension and hypertension, respectively (Gamba, 2012). We probed for differences in the membrane bound and active form of NCC (pNCC) in both the female and male hAAT-Tg and wild-type mice by Western blotting (Supplementary Figure 6). As shown in Figures 5A,B, there was significantly increased pNCC expression in female hAAT-Tg mice compared to both the male hAAT-Tg and male C57B/6 wild-type mice.

### Decreased ENaC Alpha and Increased ENaC Beta and Gamma Subunit Protein Expression in Male hAAT-Tg Mice Compared to Male Wild Type Control

**FIGURE 6:**
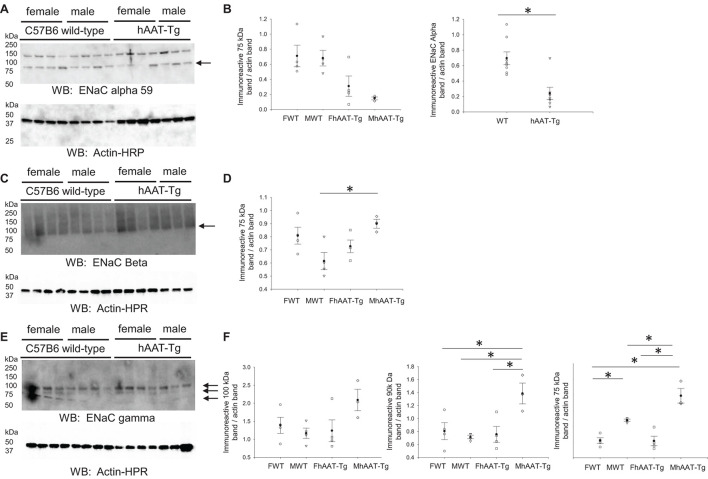
Western blot analysis of ENaC alpha, beta, and gamma protein subunit expression from the membrane fraction of kidney cortex lysates. **(A)** Western blot analysis of the alpha subunit of epithelial sodium channel (ENaC) protein expression from the membrane fraction of kidney cortex lysates harvested from female and male hAAT-Tg mice and C57B/6 wild-type mice after being maintained initially on a normal salt diet and then a high salt diet and euthanized in the AM. **(B)** Densitometric analysis of the immunoreactive ENaC alpha subunit bands in panel **(A)** normalized to actin. **(C)** Western blot analysis of the beta subunit of epithelial sodium channel (ENaC) protein expression from the membrane fraction of kidney cortex lysates harvested from female and male hAAT-Tg mice and C57B/6 wild-type mice after being maintained initially on a normal salt diet and then a high salt diet and euthanized in the AM. **(D)** Densitometric analysis of the immunoreactive ENaC beta subunit bands in panel **(C)** normalized to actin. **(E)** Western blot analysis of the gamma subunit of epithelial sodium channel (ENaC) protein expression from the membrane fraction of kidney cortex lysates harvested from female and male hAAT-Tg mice and C57B/6 wild-type mice after being maintained initially on a normal salt diet and then a high salt diet and euthanized in the AM. **(F)** Densitometric analysis of the immunoreactive ENaC gamma subunit bands in panel E normalized to actin. These results were analyzed using a One-Way ANOVA followed by a Holm-Sidak comparison and a Student’s *t*-test. We used a significance level of *p < 0.05.

ENaC is also expressed in the aldosterone sensitive distal nephron and it contributes to the fine-tuning of sodium reabsorption in the distal nephron (Pochynyuk et al., 2008). We probed for the alpha subunit of renal ENaC since this subunit is activated by proteolysis and is necessary for efficient channel activity (Alli et al., 2012b; Supplementary Figure 7). Membrane expression of the cleaved and active 75 kDa ENaC alpha protein was significantly lower in hAAT-Tg mice compared to C57B/6 wild-type control mice (Figures 6A,B). When we probed for the beta and gamma subunits of ENaC, we found significant increases in the expression of the 75 kDa ENaC beta protein in the male hAAT-Tg mice as opposed to the male control mice (Figures 6C,D) and significant increases in the expression of both the 90 and 75 kDa ENaC gamma protein in the male hAAT-Tg mice when compared to the control mice (Figures 6E,F).

### Reduced NPRC Protein Expression in hAAT-Tg Mice Compared to Wild-Type Control

**FIGURE 7:**
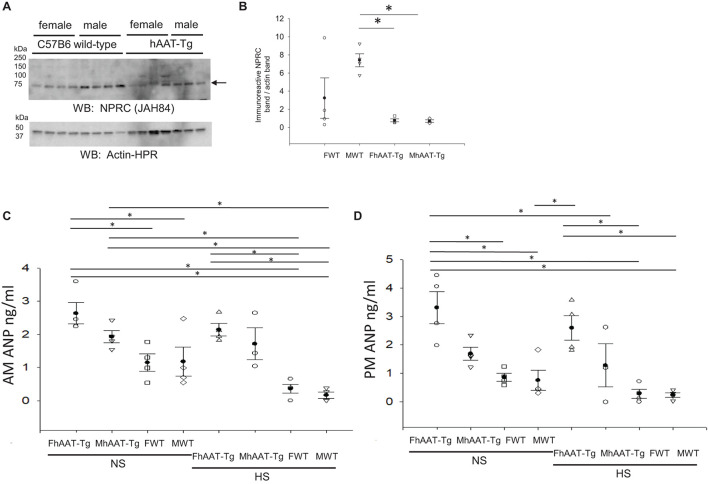
Western blot analysis of NPRC protein expression from the membrane fraction of kidney cortex lysates and blood ANP concentration analysis. **(A)** Western blot analysis of the natriuretic peptide receptor C (NPRC) protein expression from the membrane fraction of kidney cortex lysates harvested from female and male hAAT-Tg mice and C57B/6 wild-type mice. **(B)** Densitometric analysis of the immunoreactive NPRC band in panel **(A)** normalized to actin. **(C)** Atrial natriuretic peptide (ANP) concentrations in the blood of female and male hAAT-Tg mice and C57B6 wild-type mice in the inactive cycle (AM). **(D)** Atrial natriuretic peptide (ANP) concentrations in the blood of female and male hAAT-Tg mice and C57B6 wild-type mice in the active cycle (PM). *N* = 7 hAAT-Tg (4 female and 3 male) and *N* = 8 C57B/6 (4 female and 4 male) mice maintained initially on a normal salt diet and then a high salt diet. These results were analyzed using a One-Way ANOVA followed by a Holm-Sidak comparison. We used a significance level of *p < 0.05.

Natriuretic peptide receptor C (NPRC) is the clearance receptor for natriuretic peptides, and it is abundantly expressed in many tissues including the kidney (Matsukawa et al., 1999). Natriuretic peptide receptor C may regulate renal ENaC at multiple levels. First, it may attenuate the inhibition of ENaC by ANP by internalizing the peptide and reducing its concentration in the systemic circulation (Guo et al., 2013). Second, it may reduce the open probability of ENaC by the activation of PLC and subsequent hydrolysis of PIP_2_ (Tuna et al., 2019). Therefore, we investigated whether NPRC protein expression in the membrane fractions of the kidney cortex is altered in hAAT-Tg mice compared to C57B/6 mice (Supplementary Figure 10). As shown in Figures 7A,B, NPRC protein expression in the membrane fractions was significantly less in the hAAT-Tg mice compared to the C57B/6 mice.

### Greater Circulating ANP in hAAT-Tg Mice Compared to C57B/6 Wild-Type Mice

Since NPRC protein expression was attenuated in the hAAT-Tg mice compared to the wild-type mice, we investigated whether there was a difference in the circulating levels of ANP between the groups (Supplementary Figure 11). ANP levels in the blood were significantly greater in female hAAT-Tg mice compared to wild-type control mice maintained on a normal salt diet and significantly greater in male hAAT-Tg mice compared to wild-type mice maintained on a high salt diet for the inactive cycle (Figure 7C). For the active cycle, female hAAT-Tg mice maintained on either a normal salt or high salt diet had significantly greater ANP levels compared to wild-type control mice (Figure 7D).

### Analysis of NO Concentrations in hAAT-Tg and Wild-Type Mice

**FIGURE 8:**
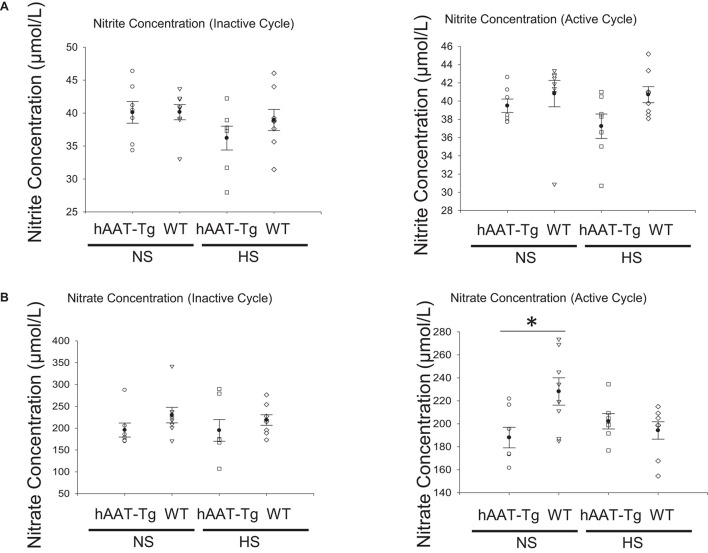
Blood nitrite and nitrate concentration analysis. **(A)** Nitrite concentrations in the blood of female and male hAAT-Tg mice and C57B/6 wild-type mice in the inactive cycle (AM) or active cycle (PM). **(B)** Nitrate concentrations in the blood of female and male hAAT-Tg mice and C57B/6 wild-type mice in the inactive cycle (AM) or active cycle (PM). These results were analyzed using a One-Way ANOVA followed by a Holm-Sidak comparison. We used a significance level of *p < 0.05.

We measured nitrites and nitrates as a measure of nitric oxide in the blood of hAAT-Tg and wild-type mice maintained on a normal or high salt diet (Supplementary Figure 12). hAAT transgenic mice had significantly less circulating levels of nitrate compared to C57B/6 wild-type mice maintained on a normal salt diet (Figures 8A,B).

## Discussion

We previously showed renal ENaC is cleaved and activated by the cysteine protease cathepsin B (Alli et al., 2012b). Cathepsin B was shown to increase ENaC activity leading to the development of hypertension in nephrotic syndrome (Larionov et al., 2019). Cathepsin B is also responsible for cleaving the myristoylated alanine-rich C kinase substrate (MARCKS) (Spizz and Blackshear, 1997), an adaptor protein that potentiates the interaction between anionic phospholipid phosphates and ENaC (Alli et al., 2012a, 2015; Montgomery et al., 2017). Anionic phospholipid phosphates such as PIP_2_ directly bind to the amino and carboxy terminal domains of ENaC subunits to keep the channel in an open confirmation (Yue et al., 2002; Ma and Eaton, 2005). Geraghty et al. (2008) first showed cathepsin B activity is inhibited by AAT in human bronchoalveolar lavage fluid. However, little is known about the regulation of cathepsin B by AAT in the kidney.

Here we investigated for the first time hAAT expression during the inactive and active cycles of female and male hAAT transgenic mice. Interestingly, we found hAAT expression is higher in female hAAT-Tg mice during the active cycle compared to inactive cycle of the same female mice. Since we observed differences in hAAT between female and male mice, we further investigated differences in proteins directly and indirectly regulated by AAT in this study.

In addition to cathepsin B, we also investigated the regulation of kidney kallikrein in male and female hAAT-Tg and C57B/6 wild-type control mice since this protease is also involved in the proteolysis of renal ENaC and was shown to be inhibited by AAT (Horl and Heidland, 1981; Schapira et al., 1986). We observed a decrease in cathepsin B protein activity in hAAT-Tg mice compared to wild-type control mice. Consistent with this finding, there was also a decrease in the proteolytically cleaved from of ENaC alpha protein, as previously characterized (Alli et al., 2012a,b), in the membrane kidney cortex fractions from hAAT-Tg mice compared to wild-type control mice. While the alpha subunit was reduced in hAAT overexpressing mice, the beta subunit of ENaC was increased (Figure 6).

The second messenger cGMP inhibits ENaC activity at multiple levels in the distal tubule and collecting duct. First, the binding of ANP to its preferential guanyl cyclase coupled cell surface receptor NPRA inhibits the open probability and activity of ENaC (Guo et al., 2013). Second, nitric oxide (NO) activation of its soluble guanyl cyclase receptor also leads to a decrease in ENaC activity (Helms et al., 2005; Yu et al., 2007). Although we attempted to measure endogenous soluble guanyl cyclase in hAAT-Tg and wild-type mice, the commercial antibody that we used revealed several non-specific bands and we could not include any quantification of this data. The decrease in NPRC protein expression in the kidney may in part explain the increase in ANP levels in the systemic circulation of hAAT-Tg mice maintained on either a normal salt or high salt diet (Figure 7C). The increase in ANP levels presumably inhibits ENaC activity in principal cells of the kidney.

Renal ENaC is involved in the fine-tuning of sodium reabsorption in the distal nephron and collecting duct and natriuretic peptides have been shown to inhibit this channel to promote natriuresis. Here we showed that hAAT-Tg mice have lower urinary concentrations of sodium, potassium, and chloride, as well as lower systolic blood pressure compared to C57B/6 wild-type mice. There are many epithelial transport mechanisms within the different segments of the nephron that contribute to electrolyte balance and blood pressure regulation such as the sodium potassium chloride cotransporter (NKCC2) in the thick ascending limb, NCC in the distal tubule, and ENaC in the distal tubule and collecting duct. The downregulation of a specific transport mechanism could be compensated by the upregulation of another transport mechanism and this could contribute to the decreased sodium excretion observed with reduced ENaC expression. Similarly, there are a myriad of pathways that contribute to electrolyte balance and resulting blood pressure regulation. There are also feedback mechanisms and cross-talk between these different pathways, for example natriuretic peptides and their cognate receptors contribute to natriuresis and a reduction in blood pressure. We also observed that each of the hAAT-Tg mice had hydronephrosis. Hydronephrosis is known to be associated with the development of salt-sensitive hypertension (Sparks and Susic, 1975; Susic et al., 1976). In other studies, severe hydronephrosis has been shown to be associated with lower plasma renin levels (Carlstrom et al., 2006). One limitation of our study is that we did not investigate RAS deficiency that may contribute to renal malformation. The conversion of inactive prorenin to active renin occurs in secretory granules after proteolytic processing. A few studies report that cathepsin B is the human prorenin processing enzyme (Wang et al., 1991; Shinagawa et al., 1995; Neves et al., 1996) while other studies report that it is not the processing enzyme for prorenin in mice (Gross et al., 2010; Mercure et al., 2010). This indicates that species differences exist.

It is not surprising that we did not observe differences in blood pressure for C57B/6 wild-type mice after switching the mice from a normal salt to a high salt diet since these mice are salt-resistant and are not used as a model to study the mechanism of salt-sensitive hypertension. Our results utilizing the same methodology (tail cuff measurements) was similar to other studies conducted which resulted in systolic blood pressure measurements of 101.9 ± 2.8 mmHg (Makaritsis et al., 1999). The difference in blood pressure in both the inactive and active cycles of hAAT-Tg mice compared to the C57/B6 mice was more striking for mice maintained on a normal salt diet compared to a high salt diet for both the inactive and active cycles (Figure 3). This suggests, the mice adapt to the salt loading presumably by the tubuloglomerular feedback mechanism.

The data from this study support a role for AAT in the regulation of proteases that are involved in the proteolysis and activation of renal ENaC. The use of hAAT-Tg mice represents a transgenic animal model that can be useful to investigate various indirect pathways of ENaC regulation in the kidney that are influenced by human AAT. The strategy of using a transgenic mouse model that overexpresses the human AAT gene may be more physiologically relevant to investigate the pathogenesis of human blood pressure disorders since there are multiple genes for mouse AAT instead of only one gene for human AAT. Future studies will further investigate whether AAT can also regulate ENaC degradation and recycling as well its association with the actin cytoskeleton since these are all regulated by proteases.

## Data Availability Statement

The original contributions presented in the study are included in the article/Supplementary Material, further inquiries can be directed to the corresponding author.

## Ethics Statement

The animal study was reviewed and approved by the University of Florida’s Institutional Animal Care and Use Committee.

## Author Contributions

LL, SS, and AAA designed the study. LL, MG, AE, TK, NE, HM, and AAA performed the experiments. LL, MG, AE, and AAA analyzed the data and prepared figures. All authors approved the final version of this manuscript.

## Conflict of Interest

The authors declare that the research was conducted in the absence of any commercial or financial relationships that could be construed as a potential conflict of interest.

## Publisher’s Note

All claims expressed in this article are solely those of the authors and do not necessarily represent those of their affiliated organizations, or those of the publisher, the editors and the reviewers. Any product that may be evaluated in this article, or claim that may be made by its manufacturer, is not guaranteed or endorsed by the publisher.

## Supplementary Material

Supplementary Figure 1 The levels of hAAT in the circulation of hAAT–Tg mice. Male (MhAAT-Tg, *n* = 4) and female hAAT-Tg (FhAAT-Tg, *n* = 3) was used. Male (MWT, *n* = 4) and female (FWT, *n* = 3) C57BL/6 mice was used as a wildtype control. Blood samples were taken in both active (PM) and inactive (AM) periods. The levels of hAAT were detected by hAAT specific ELISA.

Supplementary Figure 2 Tables of urinary electrolyte results and significant *p*-values corresponding to the inactive and active cycles of male and female hAAT-Tg and C57B/6 wild-type mice maintained on a normal or high salt diet. **(A)** Urinary electrolyte concentrations during the inactive and active cycles for female and male hAAT-Tg mice and C57B/6 wild-type mice maintained on a normal salt (NS) or high salt (HS) diet. **(B)** Significant *p*-values of the comparisons between the inactive and active cycles of male and female hAAT-Tg and C57B/6 wild-type mice maintained on a normal or high salt diet. These results were analyzed using a One-Way ANOVA followed by a Holm-Sidak comparison. We used a significance level of *P* < 0.05.

Supplementary Figure 3 Tables of urine osmolality results and significant *p*-values for male and female hAAT-Tg and C57B/6 wild-type mice maintained on a normal or high salt diet during the inactive and active cycles. **(A)** Urinary osmolality during the inactive and active cycles for female and male hAAT-Tg mice and C57B/6 wild-type mice maintained on a normal salt (NS) or high salt (HS) diet. **(B)** Significant *p*-values of the comparisons between the urinary osmolality during the inactive and active cycles for female and male hAAT-Tg mice and C57B/6 wild-type mice maintained on a normal salt (NS) or high salt (HS) diet. These results were analyzed using a One-Way ANOVA followed by a Holm-Sidak comparison. We used a significance level of *P* < 0.05.

Supplementary Figure 4 Tables of systolic blood pressures and significant *p*-values for male and female hAAT-Tg and C57B/6 wild-type mice maintained on a normal or high salt diet during the inactive and active cycles. **(A)** Tail cuff systolic blood pressure measurements during the inactive and active cycles for female and male hAAT-Tg mice and C57B/6 wild-type mice maintained on a normal salt (NS) or high salt (HS) diet. **(B)** Significant *p*-values of the comparisons between systolic blood pressure measurements during the inactive and active cycles for female and male hAAT-Tg mice and C57B/6 wild-type mice maintained on a normal salt (NS) or high salt (HS) diet. These results were analyzed using a One-Way ANOVA followed by a Holm-Sidak comparison. We used a significance level of *P* < 0.05.

Supplementary Figure 5 Tables of Cathepsin B activity assay results showing endogenous cathepsin B activity in kidney cortex lysates of wild-type and hAAT-Tg mice and resulting significant *p*-values. **(A)** Cathepsin B activity assay results showing endogenous cathepsin B activity in kidney cortex lysates of wild-type and hAAT-Tg mice. **(B)** Significant *p*-values of the comparisons between Cathepsin B activity assay results showing endogenous cathepsin B activity in kidney cortex lysates of wild-type and hAAT-Tg mice. These results were analyzed using a One-Way ANOVA followed by a Holm-Sidak comparison and a Student’s *t*-test. We used a significance level of *P* < 0.05.

Supplementary Figure 6 Tables of western blot analysis results and significant *p*-values of phospho-NCC protein expression from the membrane fraction of kidney cortex lysates. **(A)** Western blot analysis results of phospho-NCC protein expression from the membrane fraction of kidney cortex lysates. **(B)** Significant *p*-values of the comparisons between phospho-NCC protein expression from the membrane fraction of kidney cortex lysates. These results were analyzed using a One-Way ANOVA followed by a Holm-Sidak comparison. We used a significance level of *P* < 0.05.

Supplementary Figure 7 Tables of western blot analysis results and significant *p*-values of the alpha subunit of epithelial sodium channel (ENaC) protein expression from the membrane fraction of kidney cortex lysates. **(A)** Western blot analysis results of the alpha subunit of epithelial sodium channel (ENaC) protein expression from the membrane fraction of kidney cortex lysates. **(B)** Significant *p*-values of the comparisons between the alpha subunit of epithelial sodium channel (ENaC) protein expression from the membrane fraction of kidney cortex lysates. These results were analyzed using a One-Way ANOVA followed by a Holm-Sidak comparison and a Student’s *t*-test. We used a significance level of *P* < 0.05.

Supplementary Figure 8 Tables of western blot analysis results and significant *p*-values of the beta subunit of epithelial sodium channel (ENaC) protein expression from the membrane fraction of kidney cortex lysates. **(A)** Western blot analysis results of the beta subunit of epithelial sodium channel (ENaC) protein expression from the membrane fraction of kidney cortex lysates. **(B)** Significant *p*-values of the comparisons between the beta subunit of epithelial sodium channel (ENaC) protein expression from the membrane fraction of kidney cortex lysates. These results were analyzed using a One-Way ANOVA followed by a Holm-Sidak comparison. We used a significance level of *P* < 0.05.

Supplementary Figure 9 Tables of western blot analysis results and significant *p*-values of the gamma subunit of epithelial sodium channel (ENaC) protein expression from the membrane fraction of kidney cortex lysates. **(A)** Western blot analysis results of the gamma subunit of epithelial sodium channel (ENaC) protein expression from the membrane fraction of kidney cortex lysates. **(B)** Significant *p*-values of the comparisons between the gamma subunit of epithelial sodium channel (ENaC) protein expression from the membrane fraction of kidney cortex lysates. These results were analyzed using a One-Way ANOVA followed by a Holm-Sidak comparison. We used a significance level of *P* < 0.05.

Supplementary Figure 10 Tables of western blot analysis results and significant *p*-values of NPRC protein expression from the membrane fraction of kidney cortex lysates. **(A)** Western blot analysis results of NPRC protein expression from the membrane fraction of kidney cortex lysates. **(B)** Significant *p*-values of the comparisons between the NPRC protein expression from the membrane fraction of kidney cortex lysates. These results were analyzed using a One-Way ANOVA followed by a Holm-Sidak comparison. We used a significance level of *P* < 0.05.

Supplementary Figure 11 Tables of atrial natriuretic peptide (ANP) concentrations in the blood of female and male hAAT-Tg mice and C57B6 wild-type mice in the inactive (AM) and active (PM) cycles maintained on a normal salt (NS) or high salt (HS) diet. **(A)** ANP concentrations in the blood of female and male hAAT-Tg mice and C57B6 wild-type mice in the inactive (AM) and active (PM) cycles maintained on a normal salt (NS) or high salt (HS) diet. **(B)** Significant *p*-values of the comparisons between ANP concentrations in the blood of female and male hAAT-Tg mice and C57B6 wild-type mice in the inactive (AM) and active (PM) cycles maintained on a normal salt (NS) or high salt (HS) diet. These results were analyzed using a One-Way ANOVA followed by a Holm-Sidak comparison. We used a significance level of *P* < 0.05.

Supplementary Figure 12 Tables of nitrate concentrations in the blood of hAAT-Tg mice and C57B6 wild-type mice in the active (PM) cycle maintained on a normal salt (NS) or high salt (HS) diet. **(A)** Nitrate concentrations in the blood of hAAT-Tg mice and C57B6 wild-type mice in the active (PM) cycle maintained on a normal salt (NS) or high salt (HS) diet. **(B)** Significant *p*-values of the comparisons between nitrate concentrations in the blood of hAAT-Tg mice and C57B6 wild-type mice in the active (PM) cycle maintained on a normal salt (NS) or high salt (HS) diet. These results were analyzed using a One-Way ANOVA followed by a Holm-Sidak comparison. We used a significance level of *P* < 0.05.
