# Supplementary material for: Transgenic Mice Overexpressing Human Alpha-1 Antitrypsin Exhibit Low Blood Pressure and Altered Epithelial Transport Mechanisms in the Inactive and Active Cycles
Source: Front Physiol. 2021 Sep 22;12:710313. doi: 10.3389/fphys.2021.710313 (PMC8493122; doi:10.3389/fphys.2021.710313)
Supplement: Supplementary Figure 1 — The levels of hAAT in the circulation of hAAT–Tg mice. Male (MhAAT-Tg, n = 4) and female hAAT-Tg (FhAAT-Tg, n = 3) was used. Male (MWT, n = 4) and female (FWT, n = 3) C57BL/6 mice was used as a wildtype control. Blood samples were taken in both active (PM) and inactive (AM) periods. The levels of hAAT were detected by hAAT specific ELISA. [file Data_Sheet_1.pdf]

Figure S1

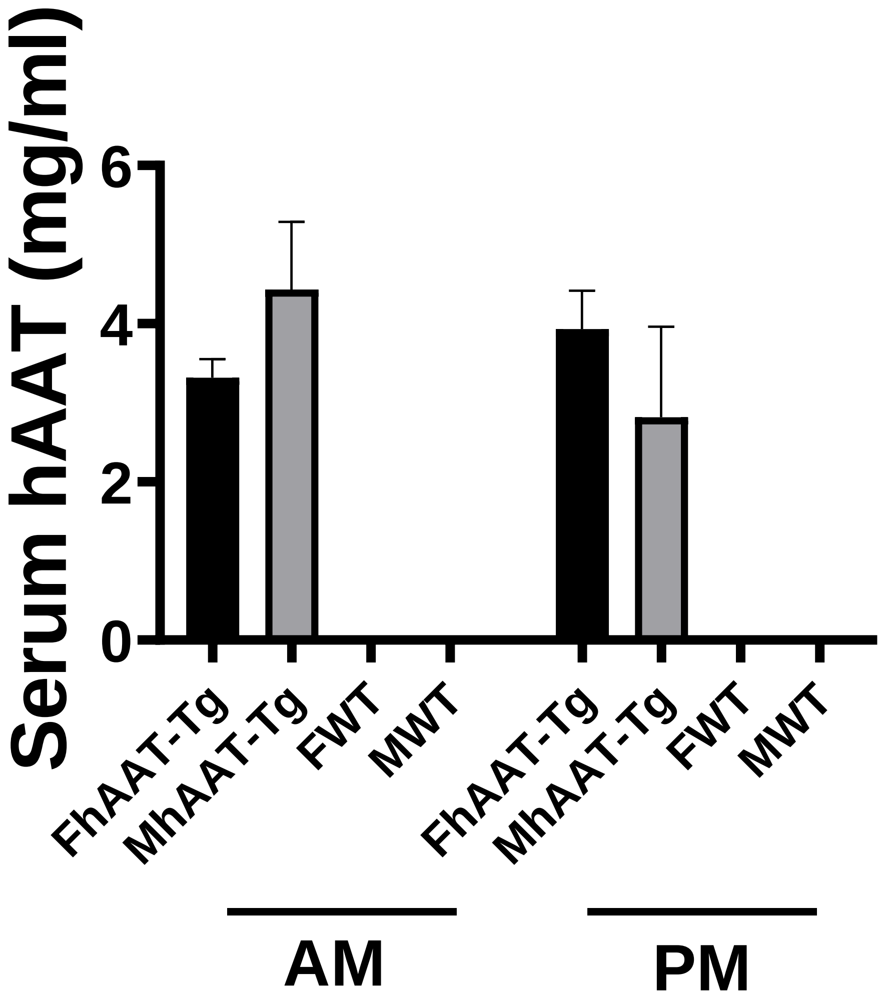

Serum hAAT (mg/ml)
6
4
2
0
FhAAT-Tg
MhAAT-Tg
FWT
MWT
FhAAT-Tg
MhAAT-Tg
FWT
MWT
AM
PM

Figure S2A

Electrolyte

A

RESULTS	NS FhAAT-Tg (n=4)	NS MhAAT-Tg (n=3)	NS FWT (n=4)	NS MWT (n=4)	HS FhAAT-Tg (n=4)	HS MhAAT-Tg (n=3)	HS FWT (n=4)	HS MWT (n=4)
**Inactive Cycle Na+ Concentration (mmol/L)**	231.42 ± 25.41	147.75 ± 47.52	560.0 ± 540.0	62.75 ± 23.42	39.55 ± 8.82	31.20 ± 4.58	381.66 ± 48.83	601.81 ± 175.36
**Active Cycle Na+ Concentration (mmol/L)**	209.14 ± 65.23	293.12 ± 72.66	72.25 ± 18.97	275.25 ± 137.91	46.50 ± 14.36	79.16 ± 10.03	601.53 ± 102.16	350.90 ± 41.02
**Inactive Cycle K+ Concentration (mmol/L)**	86.85 ± 12.16	81.75 ± 4.74	70.0 ± 14.0	109.0 ± 5.44	37.11 ± 2.45	48.80 ± 1.85	78.66 ± 6.15	62.90 ± 5.93
**Active Cycle K+ Concentration (mmol/L)**	53.71 ± 5.26	74.50 ± 10.75	91.0 ± 5.60	107.25 ± 10.05	37.0 ± 2.04	39.33 ± 5.57	79.38 ± 5.56	75.45 ± 4.86
**Inactive Cycle Cl- Concentration (mmol/L)**	194.28 ± 31.98	322.50 ± 69.84	850.0 ± 570.0	340.0 ± 33.66	464.44 ± 19.37	520.0 ± 20.0	883.33 ± 58.87	1001.81 ± 149.45
**Active Cycle Cl- Concentration (mmol/L)**	258.71 ± 85.17	370.0 ± 118.62	332.50 ± 22.01	595.0 ± 142.41	483.33 ± 23.89	513.33 ± 56.49	1007.68 ± 100.42	725.45 ± 27.24

Figure S2B

Electrolyte Significance

B

RESULTS	COMPARISONS	p-values
**Inactive Cycle Na+ Concentration**	HS MWT vs HS FhAAT-Tg	0.003
	HS MWT vs HS MhAAT-Tg	0.023
	HS MWT vs NS MhAAT-Tg	0.049
**Active Cycle Na+ Concentration**	HS FWT vs NS FWT	<0.001
	HS FWT vs HS FhAAT-Tg	<0.001
	HS FWT vs HS MhAAT-Tg	0.001
	HS FWT vs NS FhAAT-Tg	0.022
**Inactive Cycle K+ Concentration**	NS MWT vs HS FhAAT-Tg	<0.001
	NS FhAAT-Tg vs HS FhAAT-Tg	<0.001
	HS FWT vs HS FhAAT-Tg	<0.001
	NS MhAAT-Tg vs HS FhAAT-Tg	<0.001
	NS MWT vs HS MhAAT-Tg	<0.001
	NS MWT vs HS MWT	0.002
	NS FhAAT-Tg vs HS MhAAT-Tg	0.024
**Active Cycle K+ Concentration**	NS MWT vs HS FhAAT-Tg	<0.001
	NS MWT vs HS MhAAT-Tg	<0.001
	NS MWT vs NS FhAAT-Tg	<0.001
	NS FWT vs HS FhAAT-Tg	<0.001
	NS FWT vs HS MhAAT-Tg	<0.001
	HS FWT vs HS FhAAT-Tg	0.002
	HS FWT vs HS MhAAT-Tg	0.003
	HS MWT vs HS FhAAT-Tg	0.007
	NS FWT vs NS FhAAT-Tg	0.013
	HS MWT vs HS MhAAT-Tg	0.014
	NS MhAAT-Tg vs HS FhAAT-Tg	0.017
	NS MWT vs HS MWT	0.019
	NS MWT vs NS MhAAT-Tg	0.028
	NS MhAAT-Tg vs HS MhAAT-Tg	0.027
	NS MWT vs HS FWT	0.040
**Inactive Cycle Cl- Concentration**	HS MWT vs NS FhAAT-Tg	<0.001
	HS MWT vs NS MhAAT-Tg	<0.001
	HS FWT vs NS FhAAT-Tg	<0.001
	HS FWT vs NS MhAAT-Tg	0.002
	HS MWT vs HS FhAAT-Tg	0.002
	HS MWT vs NS MWT	0.004
	HS FWT vs HS FhAAT-Tg	0.030
	HS FWT vs NS MWT	0.031
	HS MWT vs HS MhAAT-Tg	0.048
**Active Cycle Cl- Concentration**	HS FWT vs NS FhAAT-Tg	<0.001
	HS FWT vs NS FWT	<0.001
	HS FWT vs NS MhAAT-Tg	<0.001
	HS FWT vs HS FhAAT-Tg	0.004
	HS FWT vs HS MhAAT-Tg	0.007
	HS MWT vs NS FhAAT-Tg	0.011
	HS FWT vs NS MWT	0.018
	HS MWT vs NS FWT	0.041

Figure S3A

Osmolality

A

RESULTS	NS FhAAT-Tg (n=4)	NS MhAAT-Tg (n=3)	NS FWT (n=4)	NS MWT (n=4)	HS FhAAT-Tg (n=4)	HS MhAAT-Tg (n=3)	HS FWT (n=4)	HS MWT (n=4)
**Inactive Cycle Osmolality (mOsm)**	1567.00 ± 98.75	1238.50 ± 61.73	2646.40 ± 273.97	2450.85 ± 186.20	1162.66 ± 56.31	1712.00 ± 169.43	2266.41 ± 130.08	1947.83 ± 46.91
**Active Cycle Osmolality (mOsm)**	1572.50 ± 104.28	1306.50 ± 61.20	2446.85 ± 273.68	2590.00 ± 205.56	1018.00 ± 47.33	1175.55 ± 45.17	2035.33 ± 179.14	1719.75 ± 66.97

Figure S3B

Osmolality Significance

B

RESULTS	COMPARISONS	p-values
**Inactive Cycle Osmolality**	NS FWT vs HS FhAAT-Tg	<0.001
	NS MWT vs HS FhAAT-Tg	<0.001
	HS FWT vs HS FhAAT-Tg	<0.001
	NS FWT vs NS MhAAT-Tg	<0.001
	NS MWT vs NS MhAAT-Tg	<0.001
	HS FWT vs NS MhAAT-Tg	<0.001
	HS MWT vs HS FhAAT-Tg	<0.001
	NS FWT vs NS FhAAT-Tg	<0.001
	NS MWT vs NS FhAAT-Tg	<0.001
	NS FWT vs HS MhAAT-Tg	<0.001
	HS MWT vs NS MhAAT-Tg	0.001
	HS FWT vs NS FhAAT-Tg	0.002
	NS MWT vs HS MhAAT-Tg	0.003
	NS FWT vs HS MWT	0.010
	HS FWT vs HS MhAAT-Tg	0.015
	HS MhAAT-Tg vs HS FhAAT-Tg	0.015
**Active Cycle Osmolality**	NS MWT vs HS FhAAT-Tg	<0.001
	NS FWT vs HS FhAAT-Tg	<0.001
	NS MWT vs HS MhAAT-Tg	<0.001
	NS MWT vs NS MhAAT-Tg	<0.001
	NS FWT vs HS MhAAT-Tg	<0.001
	HS FWT vs HS FhAAT-Tg	<0.001
	NS FWT vs NS MhAAT-Tg	<0.001
	NS MWT vs NS FhAAT-Tg	<0.001
	HS FWT vs HS MhAAT-Tg	<0.001
	NS MWT vs HS MWT	<0.001
	HS MWT vs HS FhAAT-Tg	0.002
	NS FWT vs NS FhAAT-Tg	0.002
	HS FWT vs NS MhAAT-Tg	0.004
	NS FWT vs HS MWT	0.006

Figure S4A

BP

A

RESULTS	NS FhAAT-Tg (n=4)	NS MhAAT-Tg (n=6)	NS FWT (n=4)	NS MWT (n=4)	HS FhAAT-Tg (n=4)	HS MhAAT-Tg (n=3)	HS FWT (n=4)	HS MWT (n=4)
**Inactive (AM) Systolic Blood Pressure (mmHg)**	72.62 ± 2.19	79.76 ± 2.17	92.50 ± 1.84	92.75 ± 1.10	89.75 ± 7.65	96.00 ± 4.16	105.50 ± 1.70	103.75 ± 1.70
**Active (PM) Systolic Blood Pressure (mmHg)**	63.75 ± 12.35	79.50 ± 4.22	99.25 ± 1.49	97.50 ± 0.64	100.50 ± 9.77	104.66 ± 7.51	117.00 ± 1.08	113.75 ± 1.49

Figure S4B

BP Significance

B

RESULTS	COMPARISONS	p-values
**Inactive (AM) Systolic Blood Pressure**	HS FWT vs NS FhAAT-Tg	<0.001
	HS MWT vs NS FhAAT-Tg	<0.001
	HS FWT vs NS MhAAT-Tg	<0.001
	HS MWT vs NS MhAAT-Tg	<0.001
	HS MhAAT-Tg vs NS FhAAT-Tg	0.006
	NS MWT vs NS FhAAT-Tg	0.011
	NS FWT vs NS FhAAT-Tg	0.012
	HS FhAAT-Tg vs NS FhAAT-Tg	0.045
**Active (PM) Systolic Blood Pressure**	HS FWT vs NS FhAAT-Tg	<0.001
	HS MWT vs NS FhAAT-Tg	<0.001
	HS FWT vs NS MhAAT-Tg	<0.001
	HS MWT vs NS MhAAT-Tg	<0.001
	HS MhAAT-Tg vs NS FhAAT-Tg	0.003
	NS MWT vs NS FhAAT-Tg	0.006
	NS FWT vs NS FhAAT-Tg	0.007
	HS FhAAT-Tg vs NS FhAAT-Tg	0.029
	HS MhAAT-Tg vs NS MhAAT-Tg	0.0035

Figure S5

Cathepsin B Activity Assay

A

RESULTS	WT (n=8)	hAAT-Tg (n=7)
**Cathepsin B Activity Assay (RFU)**	517.25 ± 28.59	400.00 ± 27.54

Cathepsin B Activity Assay Significance

B

RESULTS	COMPARISONS	p-values
**Cathepsin B Activity Assay**	WT vs hAAT-Tg	0.012

Figure S6

pNCC

A

RESULTS	FWT (n=4)	MWT (n=4)	FhAAT-Tg (n=4)	MhAAT-Tg (n=3)
**Immunoreactive pNCC band/ actin band**	0.99 ± 0.15	0.48 ± 0.05	1.34 ± 0.30	0.34 ± 0.11

pNCC Significance

B

RESULTS	COMPARISONS	p-values
**Immunoreactive pNCC band/ actin band**	FhAAT-Tg vs MhAAT-Tg	0.017
	FhAAT-Tg vs MWT	0.023

Figure S7

ENaC Alpha 59

A

RESULTS	WT (n=8)	hAAT-Tg (n=7)
**Immunoreactive ENaC Alpha band / actin band**	0.69 ± 0.08	0.24 ± 0.07

ENaC Alpha 59 Significance

B

RESULTS	COMPARISONS	p-values
**Immunoreactive ENaC Alpha band / actin band**	WT vs hAAT-Tg	0.009

Figure S8

ENaC Beta

A

RESULTS	FWT (n=4)	MWT (n=4)	FhAAT-Tg (n=4)	MhAAT-Tg (n=3)
**Immunoreactive ENaC Beta band/ actin band**	0.80 ± 0.06	0.61 ± 0.06	0.72 ± 0.04	0.89 ± 0.03

ENaC Beta Significance

B

RESULTS	COMPARISONS	p-values
**Immunoreactive ENaC Beta band/ actin band**	MhAAT-Tg vs MWT	0.038

Figure S9

ENaC gamma

A

RESULTS	FWT (n=4)	MWT (n=4)	FhAAT-Tg (n=4)	MhAAT-Tg (n=3)
**Immunoreactive ENaC Gamma 90 kDa band/ actin band**	0.80 ± 0.13	0.71 ± 0.02	0.75 ± 0.12	1.3 ± 0.16
**Immunoreactive ENaC Gamma 75 kDa band/ actin band**	0.217 ± 0.03	0.47 ± 0.01	0.21 ± 0.06	0.78 ± 0.09

ENaC gamma Significance

B

RESULTS	COMPARISONS	p-values
**Immunoreactive ENaC Gamma 90 kDa band/ actin band**	MhAAT-Tg vs MWT	0.014
	MhAAT-Tg vs FhAAT-Tg	0.018
	MhAAT-Tg vs FWT	0.024
**Immunoreactive ENaC Gamma 75 kDa band/ actin band**	MhAAT-Tg vs MWT	<0.001
	MhAAT-Tg vs FhAAT-Tg	<0.001
	MhAAT-Tg vs FWT	0.009
	MWT vs FhAAT-Tg	0.012
	MWT vs FWT	0.009

Figure S10

NPRC

A

RESULTS	FWT (n=4)	MWT (n=4)	FhAAT-Tg (n=4)	MhAAT-Tg (n=3)
**Immunoreactive NPRC band/ actin band**	3.23 ± 2.23	7.40 ± 0.71	0.78 ± 0.16	0.70 ± 0.14

NPRC Significance

B

RESULTS	COMPARISONS	p-values
**Immunoreactive NPRC band/ actin band**	MWT vs FhAAT-Tg	0.017
	MWT vs MhAAT-Tg	0.022

Figure S11

ANP

A

RESULTS	NS FhAAT-Tg (n=4)	NS MhAAT-Tg (n=3)	NS FWT (n=4)	NS MWT (n=4)	HS FhAAT-Tg (n=4)	HS MhAAT-Tg (n=3)	HS FWT (n=4)	HS MWT (n=4)
**Inactive Cycle (AM) ANP (ng/ml)**	2.63 ± 0.32	1.92 ± 0.18	1.14 ± 0.26	1.17 ± 0.44	2.14 ± 0.18	1.17 ± 0.48	0.356 ± 0.13	0.16 ± 0.09
**Active Cycle (PM) ANP (ng/ml)**	3.30 ± 0.57	1.69 ± 0.23	0.86 ± 0.13	0.75 ± 0.35	2.59 ± 0.43	1.27 ± 0.75	0.28 ± 0.15	0.24 ± 0.08

ANP Significance

B

RESULTS	COMPARISONS	p-values
**Inactive Cycle (AM) ANP**	NS FhAAT-Tg vs HS MWT	<0.001
	NS FhAAT-Tg vs HS FWT	<0.001
	HS FhAAT-Tg vs HS MWT	0.001
	HS FhAAT-Tg vs HS FWT	0.003
	NS MhAAT-Tg vs HS MWT	0.004
	NS MhAAT-Tg vs HS FWT	0.012
	NS FhAAT-Tg vs NS FWT	0.019
	NS FhAAT-Tg vs NS MWT	0.022
	HS MhAAT-Tg vs HS MWT	0.025
**Active Cycle (PM) ANP**	NS FhAAT-Tg vs HS MWT	<0.001
	NS FhAAT-Tg vs HS FWT	<0.001
	NS FhAAT-Tg vs NS MWT	0.002
	NS FhAAT-Tg vs NS FWT	0.002
	HS FhAAT-Tg vs HS MWT	0.004
	HS FhAAT-Tg vs HS FWT	0.004
	NS FhAAT-Tg vs HS MhAAT-Tg	0.031
	HS FhAAT-Tg vs NS MWT	0.037

Figure S12

Nitrate Concentration

A

RESULTS	NS hAAT-Tg (n=7)	NS WT (n=8)	HS hAAT-Tg (n=7)	HS WT (n=8)
**Active Cycle Nitrate Concentration (µmol/L)**	188.01 ± 8.90	228.13 ± 11.92	202.199 ± 6.67	194.20 ± 7.56

Nitrate Concentration Significance

B

RESULTS	COMPARISONS	p-values
**Active Cycle Nitrate Concentration**	NS WT vs NS hAAT-Tg	0.028
